# Supplementary material for: Active site specificity profiling datasets of matrix metalloproteinases (MMPs) 1, 2, 3, 7, 8, 9, 12, 13 and 14
Source: Data Brief. 2016 Feb 22;7:299–310. doi: 10.1016/j.dib.2016.02.036 (PMC4777984; doi:10.1016/j.dib.2016.02.036)
Supplement: Supplementary file 10 — Supplementary material [file mmc10.zip › WebPICS_hMMP9_T_1%/P2prime.html]

 

PICS results


|  |  |
| --- | --- |
| **P2prime\_C**  5 in 138 sites   3.6 %    effects > 10 perc. pnts.  (vice-versa in brackets)  P2\_C: 18.6 (46.4)   P1prime\_Q: 33.5 (18.6) |  |
| **P2prime\_F**  6 in 138 sites   4.3 %    effects > 10 perc. pnts.  (vice-versa in brackets)  P3\_C: 15.3 (45.7)   P2\_H: 12.4 (12.4) |  |
| **P2prime\_H**  10 in 138 sites   7.2 %    effects > 10 perc. pnts.  (vice-versa in brackets)  P3\_V: 20.6 (15.9)   P1\_S: 19.9 (14.2)   P1prime\_M: 15.7 (26.1) |  |
| **P2prime\_I**  15 in 138 sites   10.9 %    effects > 10 perc. pnts.  (vice-versa in brackets)  P2\_F: 15.7 (39.1)   P1\_Q: 13.5 (22.4)   P1prime\_V: 11.3 (14.1)   P3prime\_N: 14.9 (32.0) |  |
| **P2prime\_L**  15 in 138 sites   10.9 %    effects > 10 perc. pnts.  (vice-versa in brackets)  P3\_A: 17.3 (19.9)   P3prime\_G: 34.6 (14.8) |  |
| **P2prime\_M**  8 in 138 sites   5.8 %    effects > 10 perc. pnts.  (vice-versa in brackets)  P2\_M: 31.0 (27.5)   P2\_V: 17.8 (14.2)   P1\_D: 18.5 (16.4)   P1prime\_V: 16.3 (10.9)   P1prime\_W: 10.3 (27.5) |  |
| **P2prime\_Q**  14 in 138 sites   10.1 %    effects > 10 perc. pnts.  (vice-versa in brackets)  P1\_H: 11.4 (39.9)   P1prime\_I: 24.1 (13.0)   P1prime\_Q: 14.9 (23.2) |  |
| **P2prime\_T**  8 in 138 sites   5.8 %    effects > 10 perc. pnts.  (vice-versa in brackets)  P1\_P: 16.3 (10.9)   P1\_W: 10.3 (27.5)   P1prime\_W: 10.3 (27.5)   P3prime\_H: 20.7 (27.5) |  |
| **P2prime\_V**  16 in 138 sites   11.6 %    effects > 10 perc. pnts.  (vice-versa in brackets)  P2\_Q: 13.0 (25.9)   P1\_N: 17.8 (28.4) |  |
